# The Signs of Real Death in the Absence of a Doctor

**Published:** 1907-04

**Authors:** Wesley E. Taylor

**Affiliations:** Atlanta, Ga.


ATLANTA

### Journal-Record of Medicine

Successor to Atlanta Medical and Surgical Journal, Established 1855, and Southern Medical Record, Established 1870.

OWNED BY THE ATLANTA MEDICAL JOURNAL CO.

Published Monthly.

*Official Organ Fulton County Medical Society, State Examining Board, Presbyterian Hospital, Etc.*

VOL. IX. APRIL, 1907. No. 1

BERNARD WOLFF, M. D., EDITOR, 319-320 Prudential Bldg.

M. B. HUTCHINS, M. D., BUSINESS MANAGER, 1015-1016 Century Bldg.

E. G. BALLENGER, M. D., ASSOCIATE EDITOR AND ASSISTANT BUSINESS MANAGER.

### ORIGINAL COMMUNICATIONS

### THE SIGNS OF REAL DEATH IN THE ABSENCE OF A DOCTOR.

By WESLEY E. TAYLOR, B. S., M. D., Atlanta, Ga.

A rich Frenchman in years gone by had a terror of being buried alive. He appreciated the fact that the signs of death are few and even these were poorly understood by many of the physicians of his day. In order to advance science along that line and thus lessen the danger of living inhumations, he left a large sum of money, at his death, with this purpose in view. The income, about $500 per year, is allowed to accumulate and every five years is given to that person who, by written document, can show the greatest material advance along this line. Such in brief is the history of the establishment of the Durael prize. Since its foundation in 1872, it has been awarded only one time, and that was in 1890 to Dr. Icard, of Marseilles, for the results of ten years of most careful and laborious experimentation. From this moment Dr. Icard has been recognized and universally acknowledged as the leader in this field of research. The resume of his work up to that time, along with the fruits of all his labors since then, has just appeared in book form and is entitled "The Signs of Real Death in the Absence of a Physician." The work has not as yet been translated, so a brief criticism of its contents may not be out of place.

The book from all points of view is intensely interesting as well as most instructive. The results of a vast amount of research and work are presented in an admirably smooth, scholarly and at the same time terse style, and smacks of the real student in every line. The scientific facts exposed by the author could all be contained in much smaller space, but, like the Germans, he puts in a lot of padding. It is only fair to him, however, to say that it all adds much to the novel-like attractiveness of his book, though we think that one hundred pages devoted to even authentic narratives of persons who have been buried alive, is rather a liberal allowance. One cannot help but admire the careful detail with which he has done his work. Faithfulness in detail always impresses one that accuracy has likewise been acquired.

Fifty or one hundred years ago, when respiration ceased, to all outward appearances, and a body became cold and stiff, and especially if no heart action was apparent at the wrist or with the stethoscope, it was pronounced dead and was buried without further ado, sometimes on the very same day, or at most on the day following. To safeguard accident or premature burial the disposal of the dead is surrounded in almost every country by considerable formality, both legally and in regard to church rites and civil custom. Nevertheless, in spite of all this, a body is frequently rushed to the churchyard on account of climate or contagious disease, within a few hours after death is supposed to have taken place. It happens, particularly out in the country where physicions are hard to get, that the condition of death has not been determined by even a competent person. Not only this, but a physician will frequently come too late and be told at the door that the patient is dead, and sign the certificate without even seeing the body. Perhaps he comes in, the room is dark, the body is dressed and laid out carefully and he hesitates to disturb the arrangements and so at most may only feel for the pulse. Not finding it, he naturally assumes that death has occurred and, passing out, signs the death certificate, and the patient is buried. Such is indeed the usual routine even in this advanced day and time, and it is to safeguard patients against such carelessness that the work of Dr. Icard was directed. He points out many aunthentic instances where death has been pronounced by physicians after examination, and where the corpse has revived spontaneously and lived many years. This return to life has taken place sometimes before the body has been placed in the coffin; sometimes it has occurred during the process of interment, and in a few instances the grave has been opened and a live person found, while in one or two extraordinary cases the dead man has returned to life and after extricating himself unaided from the grave, has told the horrible story of his sensations when he found himself buried alive. How many cases may have revived after interment, only to die by suffocating, no mortal being will ever know, but the number must be considerable from statistics presented by Dr. Icard. He has searched the literature pretty thoroughly, as well as the records of the Catholic Church, and authenticated every case he reports beyond the shadow of a doubt. The stories read almost like a dime novel.

If it is sometimes difficult for a competent physician to determine if a patient is dead, even after a careful examination by the ordinary methods, and if they sometimes make mistakes, it is necessary that some simple means be devised that can be used by one outside of the profession and which will render a mistake impossible. It must be something that no circumstance will render impractical and that will not mutilate the body or shock the relatives—something whose action is rapid and whose testimony is sure and positive, and which can be interpreted by the ignorant. It must be so simple that even careless persons cannot well make a mistake. It was toward the discovery of such a test that Dr. Icard bent his energies. 'e shall see presently how admirably he has succeeded. So simple are his tests and so convincing are their proofs that if they are carried out at all there is scarcely a possibility of error even under the most unfavorable circumstances. The only error that could be made would be one that would delay necessary, rather than cause premature burial.

The two unquestionable signs of death are permanent cessation of circulation and putrefaction. In regard to the first, even the circulation may cease for a short period of time and resume again. The heart may apparently stop for many minutes and no ear detect any action. It is said that life may be revived in animals even after twenty minutes, but that after a condition of suspended circulation has existed longer than this, recssitation has always been found impossible. Therefore the condition of suspended circulation during the brief period that a physician examines with his stethoscope does not prove that the heart may not resume in a few moments and a mistaken diagnosis result. Respiration may become so shallow as to escape observation for hours at a time or, like the heart, cease altogether for a time and resume later on. Rigor mortis is a condition likely to cause error, as there are many conditions which simulate it closely. In certain diseases and in mulattoes, the *livores mortis* are not distinguishable The temperature falls below normal in many cases where there is no death, or may rise above normal in the cadaver, or the room temperature may prevent any appreciable change.

Putrefaction is the one surely unmistakable sign of death that can be appreciated by the laity, but it is rarely visible to the naked eye before the fourth day. On this account it is impractical, since one cannot always keep a body till it makes its appearance.

There are many signs of life which are very sensitive and if properly made will prove a person alive; but failure to find them does not prove death. One or two are especially valuable, but they all require more or less experience in their application and so are impractical. The loss of elasticity of the skin, as demonstrated by pinching up a fold, if properly done, is a good sign, though not infallible, as well as is the translucency of the fingers to light. One can make an incision with a scalpel to determine whether the circulation has ceased or not, but none of these signs are satisfactory. There is still one which, if properly done, is beyond question a negative proof of death, and that is the "fluorescine test," devised by Dr. Icard himself. It requires only the necessary drug, a drachm or two of flourescine, and a hypodermic needle. Injected into a living person, the fluorescine is carried by the circulation, if present or if resumed at any later period, to all parts of the body and imparts at once a bright saffron color to the tissues and anyone can see immediately that circulation and life exist. The process is harmless, for if the patient is alive the drug is rapidly eliminated by the kidneys and no harm is done. In cases of jaundice or in cases which die soon afterwards, it is in a measure unsatisfactory. Besides, it is a measure that necessitates the presence of a physician and so is of little value in the country where he is sometimes hard to get. The author maintains that a physician should always be called to pronounce upon the existence of death, and insists that he should make a careful examination in all cases and not take it for granted because he fails to find pulse or to hear the heart with his stethoscope. Dr. Icard has, however, discovered another test or proof of death, which is not only an early one, but is simple and infallible. The presence of this sign precludes all possibility of a return to life, as it is dependent on putrefaction for its existence. Putrefaction, as he has mentioned, is the one sign, besides the permanent cessation of circulation, which is a positive sign of death. The circulation may apparently cease, as has been mentioned, and then resume again, and its detection is difficult. Putrefaction begins only when life ceases, and it is toward the earliest possible detection of this that the author bends his energies.

Decomposition begins in all probabilities first in the lungs, and it is at that point that it is most easily detected. The first products of decomposition are the sulphides of hydrogen and ammonium, which are liberated in considerable quantity from the free and moist surface of the respiratory tract, and escape from the nostrils, if the mouth be kept closed. There are no sulphurous gases found in the body during life, either in the bowel, stomach or even in fetid bronchal or lung conditions, at least sufficient to give a test for the same. After death the quantity is very large, and a test can be made and the presence of gas, as well as the absence of life or any possibility of its return proven to the naked eye of any observer.

Dr. Icard resorts to a very ingenious and simple method to detect this gas. He takes a piece of filter paper, moistened in a neutral solution of lead acetate in two parts of distilled water, and places it either in or over the nostrils. The hydrogen sulphide gas escaping and coming in contact with the lead forms black lead sulphide, blackening the paper and thus making an indellible sign of death. In the absence of the lead acetate solution, a freshly brightened piece of copper or silver money will serve very well, and the blackened or tarnished stain of silver or copper sulphide is left upon the coin. These signs are easily obtainable within from twelve to thirty-six hours after death, according to climatic conditions. The author thinks that this test should be resorted to in all cases before a burial permit is given, and recommends that its observance be compelled by legal measures. In this way, there would be no possibility of living interments in the future. He devotes several pages to the discussion of this feature of the case from the standpoint of the French laws, which will not interest us.

On the whole, the book is a most readable and instructive one, and worthy of translation and a place in any library.

